# Maternal Quality of Life before and after Delivery

**DOI:** 10.5812/ircmj.3646

**Published:** 2013-07-05

**Authors:** Seyed Ebrahim Ahmadi, Ramin Mozafari, Afsaneh Azari, Mohammad Reza Nateghi

**Affiliations:** 1 Iranian Biological Recourse Center, Tehran University of Medical Sciences, Tehran, IR Iran; 2 Child Health Research Center, Tehran University of Medical Sciences, Tehran, IR Iran

**Keywords:** Quality of Life, Hospitals, Maternity, Fertility

*Dear Editor,*

Infertility is a real crisis of life, which leads to depression, anxiety, social isolation and sexual disorder. The people, who look for the treatment of their infertility, are more anxious and worried than other people in the society (1). Every year, more than one million children are born all over the world, by these assisted reproductive techniques (2). Another factor is added to the indices of mortality, birth, outbreak and onset of the disease, and that`s the quality of life (3).

The quality of life means the people`s perception of their own position in life from the view point of culture, the value system they live in, purposes, norms, expectations and priorities. The quality of life is a personal affair, based on the person`s perception of different aspects of life (4). This research is a Cross-sectional study. 146 infertile women, being pregnant through the assisted reproductive techniques in the Royan Institute, were selected as the case group, and also 130 fertile women, being normally pregnant, were selected among those visited by gynecologists, as the control group.

All of these women were experiencing their first pregnancy; they were residents of Tehran, and between 20 – 35 years old. After exerting the exclusion criteria, including the postpartum depression, disability, or uncontrolled disease of the mother or baby, multiple birth, stillbirth and the newborn`s death, about 86 infertile mothers and 76 fertile mothers answered the Persian questionnaire about the quality of life (SF 36), which was standardized in the Research Institute of Health Sciences, to be applied in Iran, not only through the last three months of pregnancy, but also after the first month of delivery. To study the difference between the fertile and infertile mothers` quality of life before and after delivery, independent sample t-test and paired t-test were applied.

The results show that there is no difference between the fertile and the infertile mothers` quality of life during their pregnancy period (P = 0.17), but after the delivery, the average quality of life in fertile mothers will be higher than infertile mothers (P = 0.02). Also the average quality of life in fertile mothers, after the delivery is higher than that before the birth (P = 0.04). The average quality of life in infertile mothers after delivery, is also higher (P < 0.001) than that in their pregnancy period (Table 1).The changes in the quality of life of the mothers of the children born through infertility treatments, comparing to fertile mothers, has increased after the delivery (P = 0.03). As a result of this research, the quality of life in pregnant mothers either fertilized normally or through assisted reproductive techniques, did not show a significant difference during the third three months of pregnancy. In a survey carried out in Valiye-asr reproductive health institute, the results revealed that the pregnancy chance in unfertilized persons, suffering less stress and depression, is more than the others, (5) this might have led to the improvement of their quality of life.

After delivery, the quality of life in normally pregnant women is significantly better than during pregnancy (P = 0.04). In a research published in 2005, the quality of life was studied in about 29 healthy pregnant mothers, and the results showed that as the mother approaches the delivery time, her quality of life decreases, but after the delivery, this quality improves (6). The quality of life in pregnant mothers who are fertilized through fertility-assistant methods, was significantly higher after the delivery, and was even higher than the quality of life in fertilized mothers after their delivery (P = 0.04). As a whole, the improvement in the quality of life in mothers, pregnant through the fertilization-assistant method, was higher than that of the normally fertilized mothers. The reason probably was due to the removing of the infertility mark, they had to tolerate before, since the evidences indicate that their infertility deeply and emotionally affects their life (7). Paying attention to the aspects of the infertile mothers` quality of life and also planning to upgrade that, may lead to the upgrade of the infertile couples` health level, consequently there is the necessity for more investigations into the men and women`s quality of life.

**Table 1.** The Quality of Life in Normal Pregnancy and Infertile Pregnancy Group Before and After Delivery

	Infertile Pregnancy, Mean ± SD	Normal Pregnancy, Mean ± SD	
**Before the delivery**	57.95 ± 17.69	61.71 ± 17.46	0.17
**After the delivery**	74.31 ± 16.44	68.39 ± 17.10	0.02
**P Value**	< 0.001	0.04	
